# Aquaporin-3 and Aquaporin-4 Are Sorted Differently and Separately in the Trans-Golgi Network

**DOI:** 10.1371/journal.pone.0073977

**Published:** 2013-09-18

**Authors:** Eva C. Arnspang, Sabrina Sundbye, W. James Nelson, Lene N. Nejsum

**Affiliations:** 1 Department of Physics, Chemistry and Pharmacy, MEMPHYS-Center for Biomembrane Physics&DaMBIC – Danish Molecular Biomedical Imaging Center, University of Southern Denmark, Odense M, Denmark; 2 Department of Molecular Biology and Genetics and Interdisciplinary Nanoscience Center (iNANO), Aarhus University, Aarhus, Denmark; 3 Department of Biology, Stanford University, Stanford, California, United States of America; Institute of Molecular and Cell Biology, Biopolis, United States of America

## Abstract

Aquaporin-3 (AQP3) and aquaporin-4 (AQP4) are homologous proteins expressed in the basolateral plasma membrane of kidney collecting duct principal cells, where they mediate the exit pathway for apically reabsorbed water. Although both proteins are localized to the same plasma membrane domain, it is unknown if they are sorted together in the Golgi, or arrive in the same or different vesicles at the plasma membrane. We addressed these questions using high resolution deconvolution imaging, spinning disk and laser scanning confocal microscopy of cells expressing AQP3 and AQP4. AQP3 and AQP4 were observed mostly in separate post-Golgi carriers, and spinning disk microscopy showed that most of AQP3 and AQP4 were delivered to the plasma membrane in separate vesicles. In contrast, VSV-G and LDL-R, two well-charcterized basolateral proteins, co-localized to a high degree in the same post-Golgi carriers, indicating that the differential sorting of AQP3 and AQP4 is specific and regulated. Significantly, a chimeric AQP3 containing the AQP4 cytoplasmic tails co-localized with AQP4 in post-Golgi vesicles. These results indicate that AQP3 and AQP4 are separated into different post-Golgi carriers based on different cytoplasmic domain sorting signals, and are then delivered separately to the plasma membrane.

## Introduction

Polarized epithelia separate different biological compartments in an organism and transfer ions and solutes between those compartments. In general, the apical plasma membrane faces the outside of the organism, and the basal and lateral plasma membranes adhere to the extracellular matrix and neighboring cells, respectively, and collectively face the serosa. Specific channels and transporters are localized to either the apical or the basolateral membrane domain and regulate vectorial transport of water, ions and nutrients between these compartments (for review see [1]).

To maintain functional cell polarity, newly-synthesized plasma membrane proteins are delivered to the correct plasma membrane domain. Furthermore, the number and type of newly synthesized proteins delivered to the plasma membrane is regulated to maintain homeostasis in response to physiological challenges. Considerable evidence has accumulated that in most cells apical and basolateral proteins are sorted from each other in the TGN [2] from which they exit in separate carriers and are transported to their target membrane (reviewed in [3]). In addition, some proteins pass through the recycling endosomes en route to the plasma membrane (reviewed in [4]). Apical proteins are thought to be sorted in the *trans-*Golgi Network (TGN) via lipid rafts and delivered directly to the apical membrane (reviewed in [3], [5]).

Aquaporins (AQPs) are channels that facilitate the passive transport of water across a membrane by an osmotic gradient (reviewed in [6]). In the renal collecting duct, vasopressin mediates fine-tuning of urine concentration via regulation of AQP2. Short-term regulation includes AQP2 re-localization from sub-apical intracellular vesicles to the apical plasma membrane, instantly increasing water influx and, thus, the concentration of urine [7]. The exit pathway for water is thought to be mediated by AQP3 and AQP4, [8], [9] which are localized to the basolateral membrane. Gene knock-out of AQP3 induces nephrogenic diabetes insipidus and an inability to concentrate urine [10], whereas AQP4 gene knock-out produces a mild urinary concentration defect [11]. However, in isolated inner medullary collecting ducts (IMCDs) water permeability of AQP4 knockout IMCD was reduced four-fold compared to wild type [12]. AQP3 expression levels were unchanged, indicating a lack of compensation by up-regulation of AQP3 [12]. Moreover, upon long-term stimulation with vasopressin, AQP3 mRNA and protein levels increased, whereas those of AQP4 remained unchanged [13]. These results raise the possibility that AQP3 and AQP4 are regulated independently in the biosynthetic pathway.

It is unknown if different proteins destined for the basolateral membrane domain are mixed together in a common carrier in the TGN, or are sorted into separate carriers. A single common carrier might indicate a default pathway for exit of all basolateral proteins. However, multiple different carriers might indicate that the TGN controls the types of basolateral proteins that are co-sorted together into vesicles and hence would be subject to co-ordinate regulation in their delivery to the plasma membrane. Other studies have indicated that some basolateral proteins take different intracellular pathways to the plasma membrane although the site and mechanism involved in protein sorting were not identified [14], [15]. Here, we tested whether AQP3 and AQP4 are sorted into the same or different post-Golgi carriers that are delivered together or separately to the plasma membrane. We provide evidence that AQP3 and AQP4 are sorted from each other in the TGN based on different cytoplasmic domain sorting signals.

## Results

### AQP3 and AQP4 sorting in the TGN

Sorting of AQP3 and AQP4 in the TGN was analyzed in MDCK cells stably co-expressing AQP3-EGFP and Orange-AQP4, and by immunofluorescence of MDCK cells transiently co-expressing untagged AQP3 and AQP4. Newly synthesized proteins were accumulated in the TGN by a 19°C temperature block in the presence of cyclohexamide (Figure S1), and released from the temperature block by a 10 min incubation at 37°C, which was the time-point for observing most post-Golgi carriers. The distribution of proteins in the TGN and post-Golgi carriers in fixed cells was analyzed with a Zeiss LSM 510 confocal microscope, a Nikon spinning disk microscope and a DeltaVision deconvolution microscope. For deconvolution, all images and channels were processed automatically and identically. Manual post-processing was applied only to adjust the brightness and contrast of the original images to equal intensities; overall, this processing removed out-of-focus fluorescence and sharpened the contours of vesicles (see Figure S2 for example).

**Figure 1:**
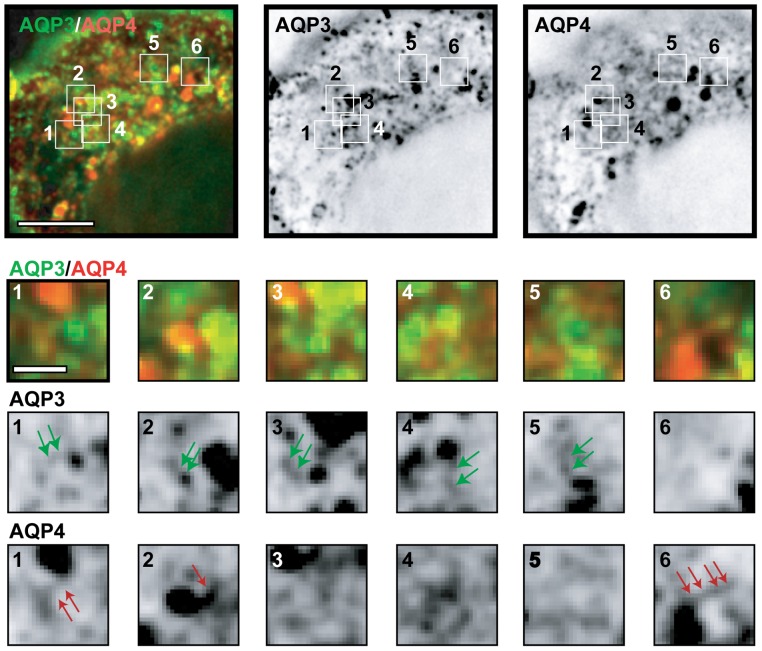
AQP3 and AQP4 are separated in post-Golgi carriers. Representative example of a single plane of a deconvolved image of a single cell stably expressing AQP3-EGFP and Orange-AQP4 10 minutes following release from a 19°C temperature block and inserts of the marked areas: green arrows point to AQP3-EGFP containing TGN and post-Golgi carriers, red arrows point to Orange-AQP4 containing TGN and post-Golgi carriers. Scale bars are: 5 μm (overlay and single channel); inserts: 0.5 μm.

**Figure 2:**
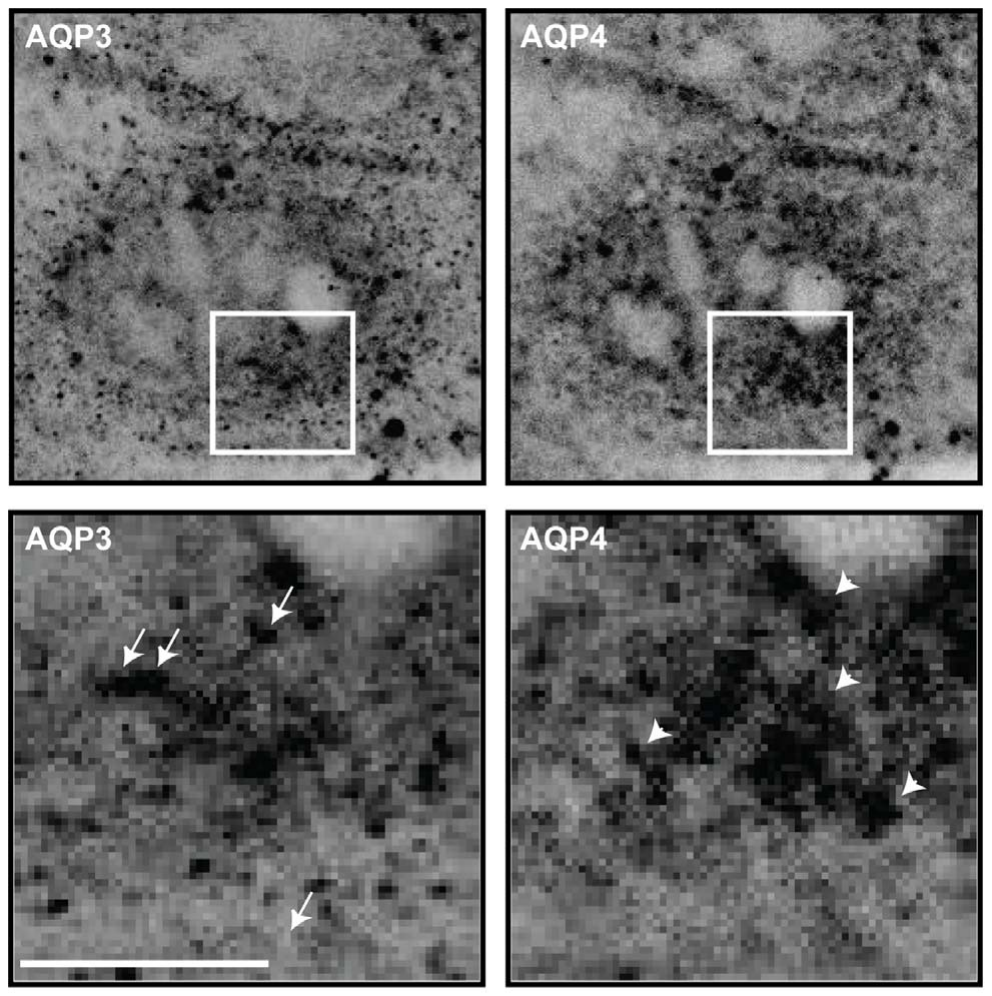
AQP3 and AQP4 are separated in post-Golgi carriers. Representative example of a single plane of a cell transiently expressing AQP3-IRES-EGFP and AQP4-IRES-EGFP labeled with antibodies towards AQP3 and AQP4, 10 minutes following release from a 19°C temperature block and inserts of the marked areas: arrows point to AQP3 containing TGN and post-Golgi carriers, arrowheads point to AQP4 containing TGN and post-Golgi carriers. Scale bars are 5 μm.

AQP3-EGFP and Orange-AQP4 localized in the TGN and in vesicular carriers leaving the TGN. We identified many examples in which AQP3-EGFP and Orange-AQP4 appeared to be separated into different regions of the TGN (Figure 1), and fewer areas in which they appeared to overlap (Figure 1; yellow, overlay; see Figure S3 for an example captured by spinning disk microscopy, Figure S4 for z-view, and Figure S5 for time 0 min). To test if the same localization pattern was observed with untagged AQP3 and AQP4, MDCK cells were transiently transfected with AQP3-IRES-EGFP and AQP4-IRES-EGFP followed by the 19°C temperature block and release as described above, and then processed for immunofluorescence microscopy. These images revealed that AQP3 and AQP4 were in separate regions of the TGN, and were present in different vesicular carriers (Figure 2). We also examined the distribution of AQP3-EGFP and Orange-AQP4 in fully polarized MDCK cells grown on Transwell filters (Figure S6). We identified cytoplasmic structures that contained either AQP3-EGFP or Orange-AQP4, and only a few that contained both. These images indicate that AQP3-EGFP and Orange-AQP4 are sorted into separate transport vesicles in non-polarized MDCK cells. Since the expression levels of AQP3-EGFP and Orange-AQP4 were low in fully polarized MDCK cells on filters, we were unable to unambiguously identify post-Golgi vesicular carriers, and therefore we focused a more detailed analysis of protein sorting in the TGN of single MDCK cells.

**Figure 3:**
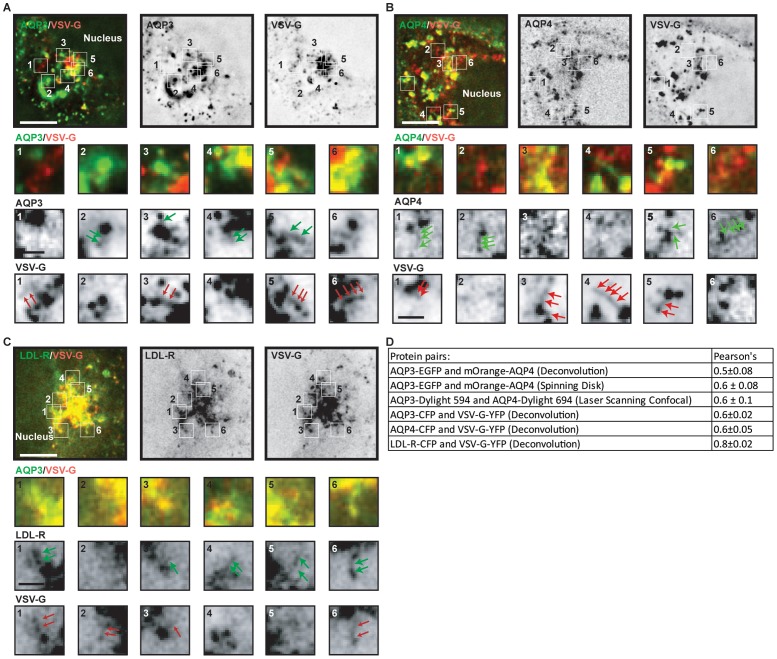
TGN localization of different pairs of basolateral proteins. (A–C) Representative examples of a single plane of deconvoluted images of single cells expressing (A) AQP3-CFP and VSV-G-YFP, (B) VSV-G-YFP and CFP-AQP4 and, (C) LDL-R-CFP and VSV-G-YFP 10 minutes following release from a 19°C temperature block. Arrows in inserts point to TGN and post-Golgi carriers. Scale bars are: 5 μm (overlay and single channel); inserts 0.5 μm. (D) Pearson's coefficient ± SEM for the different protein pairs.

We measured the overlap in the distributions of AQP3-EGFP and Orange-AQP4, and AQP3-Dylight 549 and AQP4-Dylight 649 in optical sections of the TGN region (for example, see Figures 1 and 2 from 4–5 different cells using Pearson's Correlation, a statistical method that measures the degree to which two variables are related; see Materials and Methods); note that a Pearson's coefficient of 0 defines no correlation, +1 a perfect positive correlation, and −1 a perfect negative correlation. AQP3-EGFP and AQP4-Orange were detected by deconvolution microscopy and spinning disk microscopy. AQP3-Dylight 549 and AQP4-Dylight 649 were detected using laser scanning microscopy. The Pearson's coefficient for AQP3-EGFP and Orange-AQP4, and AQP3-Dylight 549 and AQP4-Dylight 649 was 0.6 and 0.5, respectively, by deconvolution microscopy (Figure 3D), indicating little overlap in their distributions. Higher magnification images (Figures 1 and 2, inserts) revealed single post-Golgi vesicular carriers close to the TGN. These vesicular carriers generally contained either AQP3-EGFP (Figure 1 inserts, middle row, green arrows and Figure 2 inserts, arrows) or Orange-AQP4 (Figure 1 inserts, bottom row, red arrows, and Figure 2 inserts, arrowheads). Carriers containing both proteins were less frequently observed. These results indicate that AQP3 and AQP4 are sorted into different post-Golgi vesicular carriers in the TGN.

To test if sorting of these different basolateral AQPs is specific to this class of membrane proteins or a more general feature of basolateral membrane proteins, we investigated two other well characterized basolateral proteins, LDL-R and VSV-G. We examined pair-wise expression of VSV-G-YFP and either AQP3-CFP or AQP4-CFP, and LDL-R-CFP and VSV-G-YFP after release from the 19°C temperature block. We found that VSV-G-YFP and AQP3-CFP (Figure 3A), and VSV-G-YFP and CFP-AQP4 (Figure 3B) also had different distributions in the TGN with little overlap (yellow, Figure 3A and 3B), and a corresponding low Pearson's coefficient of 0.6 (Figure 3D). In addition, we identified individual post-Golgi vesicular carriers that appeared to contain only one of the proteins, but rarely both (Figure 3A, B; magnified inserts).

In contrast, we found that LDL-R-CFP and VSV-G-YFP more closely co-localized in the TGN, and we could identify many vesicular carriers close to the TGN that contained both proteins (Figure 3C, magnified inserts). The Pearson's coefficient for LDL-R-CFP and VSV-G-YFP was correspondingly higher (0.8; Figure 3) than that of the other protein pairs analyzed (see above). Thus, some basolateral proteins sort to different post-Golgi vesicular carriers, and some to the same vesicular carriers.

### Importance of the cytoplasmic domains of AQP3 and AQP4 for AQP sorting into different post-Golgi carriers

As sorting of basolateral proteins into post-Golgi carriers is mediated by cytoplasmic sorting signals [16], we generated a chimeric protein in which the cytoplasmic domain of AQP3 was substituted for that of AQP4, so that the chimeric protein had the structure and function of AQP3 but the cytoplasmic tail domain of AQP4 (Figure S7A; GFP-AQP434). Importantly, GFP-AQP434 localized correctly to the basolateral plasma membranes of polarized MDCK cells on Transwell filters (Figure S7B).

A caveat of using GFP-AQP434 to examine protein sorting is the potential for hetero-tetramerization of chimeric proteins with endogenous AQPs in MDCK cells. However, aquaporins do not heterotetramerize (for review see [6]), and MDCK cells do not express AQP3 at significant levels until they reach confluency [17], thus hetero-tetramerization of endogenous AQP3 with GFP-AQP434 should not occur.

**Figure 4:**
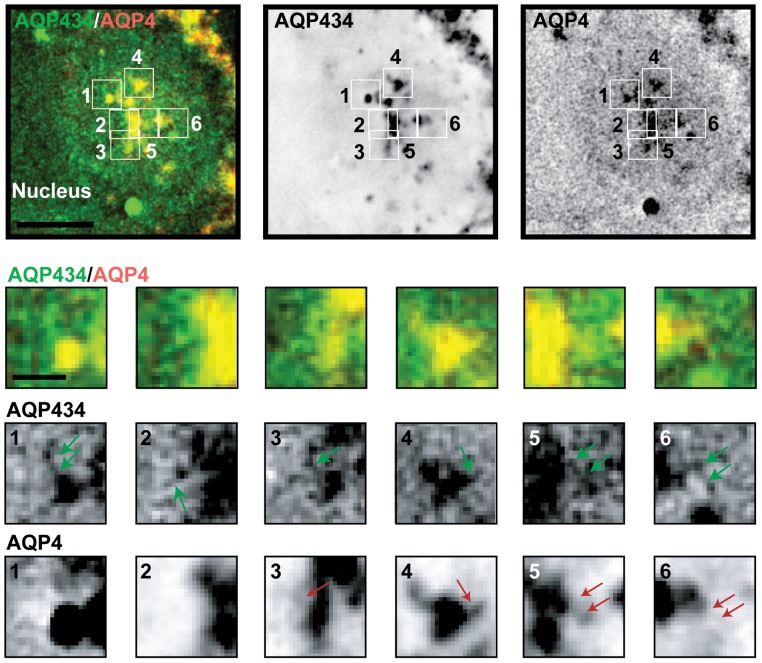
AQP4 and AQP434 co-localize in post-Golgi carriers. Representative example of a single plane of a deconvolved image of a single cell expressing GFP-AQP434 and Orange-AQP4 10 minutes following release from a 19°C temperature block, and inserts of the marked areas: green arrows point to GFP-AQP434 containing post-Golgi carriers, red arrows point to Orange-AQP4 containing TGN and post-Golgi carriers. Scale bars are A: 5 μm (overlay and single channel); inserts: 0.5 μm.

Co-expression of Orange-AQP4 with GFP-AQP434 revealed a higher degree of co-localization of these proteins in the TGN and post-Golgi vesicular carriers (Figure 4), compared to AQP3-GFP and Orange-AQP4 (Figure 1). The Pearson's coefficient for Orange-AQP4 vs. GFP-AQP434 was accordingly higher than that of AQP3-GFP vs. Orange-AQP4 (0.8 vs. 0.5). These results indicate that replacing the cytoplasmic tail of AQP3 with that of AQP4 caused increased sorting of chimeric AQP3 into TGN carriers that contained AQP4. These results demonstrate that sorting signals in the cytoplasmic domain, rather than the identity of the protein itself, affect whether two different AQPs exit the Golgi in the same or a different carrier vesicle.

### Differences in the contents of post-Golgi carriers are maintained between the Golgi and plasma membrane

Although AQP3 and AQP4 proteins appear to exit the TGN in different post-Golgi carriers, we further tested whether they arrived separately or together at the plasma membrane. This analysis is complicated by the finding that some basolateral proteins transit through the recycling endosome *en route* to the plasma membrane [15], [18]. In the recycling endosome, protein-specific post-Golgi carriers may merge or be sorted again, and exit either in common carriers or specific carriers destined for the plasma membrane.

**Figure 5:**
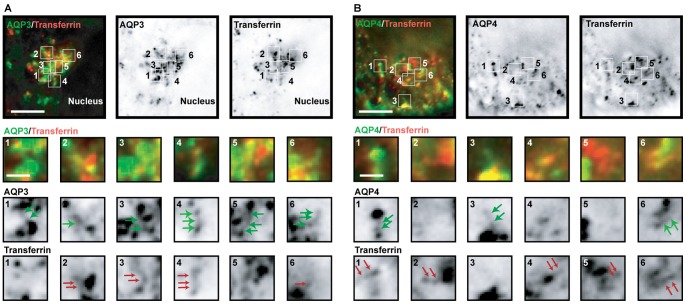
AQP3 and AQP4 do not significantly co-localize with transferrin. (A and B) Representative examples of a single plane of deconvolved images of single cells loaded with Texas-Red transferrin (red) stably expressing (A) AQP3-EGFP and (B) EGFP-AQP4 10 minutes following release from a 19°C temperature block: arrows in inserts point to post-Golgi carriers. Scale bars are 5 μm (overlay and single channels); 0.5 μm (inserts).

We examined whether AQP3-EGFP and EGFP-AQP4 merged with the recycling endosome following exit from the TGN. Cells were incubated with Texas-Red labeled Transferrin to label the recycling endosome prior to the 19°C temperature block, and AQP3-EGFP and EGFP-AQP4 were imaged 10 min following release from the 19°C temperature block. Deconvolution imaging revealed little overlap of AQP3-EGFP (Figure 5A) or EGFP-AQP4 (Figure 5B) with Texas-Red labeled Transferrin in the recycling endosome; the Pearson's coefficient was 0.5±0.1 for both. Since, we were unable to continuously image live cells, however, it remains possible that both proteins transited through the recycling endosome prior to their delivery to the plasma membrane, and that the Pearson's coefficient reflects the summation of proteins at different stages of delivery to, transit through, and exit from the recycling endosome.

**Figure 6:**
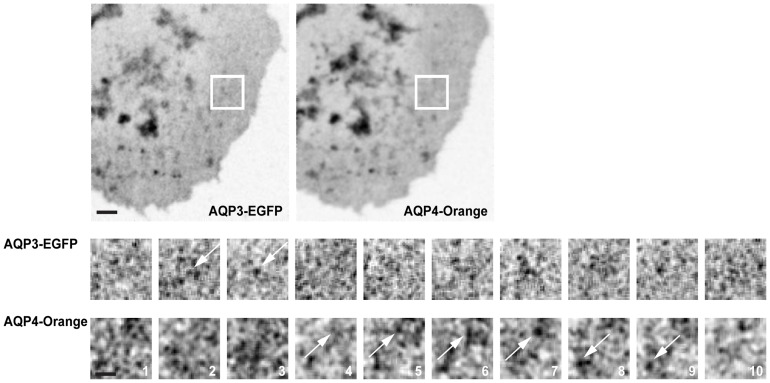
Post-Golgi transport carriers containing AQP3-EGFP and Orange-AQP4 arrived separately at the plasma membrane. Representative example of time-lapse images captured by Spinning Disk Microscopy of a single cells stably expressing AQP3-EGFP and Orange-AQP4, 10 minutes following release from a 19°C temperature block. Top panel shows an average of 10 frames. Bottom panel shows magnified inserts from 10 consecutive time frames from the indicated area. Arrows point to vesicular structures. Scale bars are 2 μm (top panel) and 1 μm (bottom panel). Images were captured every 40 mSec.

To test whether post-Golgi transport carriers containing AQP3-EGFP and Orange-AQP4 arrived separately at the plasma membrane, we used spinning disk confocal live-cell microscopy. Images were captured in pseudo-Total Internal Reflection Fluorescence (TIRF) mode in very close proximity to the plasma membrane. We could identify many examples of vesicles coming into close proximity with the basal plasma membrane that contained either AQP3-EGFP or Orange-AQP4 (Figure 6), but not both. These results indicate that AQP3 and AQP4 were sorted and exited the TGN in separate carriers, and reached the plasma membrane in separate carriers.

## Discussion

The majority of epithelial channels, transporters and receptors in polarized epithelial cells are localized to either the apical or basolateral plasma membrane domains, and correct sorting and delivery of these plasma membrane proteins is essential for maintaining homeostasis. It is well known that proteins destined for the apical and basolateral plasma membrane domains are separated in the TGN and take different routes to the plasma membranes (reviewed in [3]). However, it is less clear whether proteins destined for the same plasma membrane domain take a common sorting pathway in the TGN and delivery to the plasma membrane, or if multiple TGN sorting and transport pathways exist. These different scenarios may have physiological implications for mechanisms that coordinate the protein content of the plasma membrane for cellular regulation of homeostasis in response to physiological signals. Since AQP3 and AQP4 are differentially regulated upon long-term vasopressin stimulation [13], we tested how AQP3 and AQP4 are sorted in the biosynthetic pathway.

We found that AQP3 and AQP4 appear to exit the TGN in different post-Golgi vesicular carriers, while other basolateral membrane proteins exited the TGN in the same vesicular carriers. Using AQP3 and AQP4 and a chimeric AQP434, we uncovered two properties for sorting of AQP3 and AQP4 in the TGN. First, sorting does not appear to be based on either structural or functional homology between proteins, as AQP3 and AQP4 are highly homologous proteins and exit the TGN in different vesicular carriers. In contrast, two structurally and functionally different proteins, LDL-R and VSV-G, exited in common vesicular carriers. Second, TGN sorting appears to be determined by the cytoplasmic tail of AQP3 and AQP4, since exchange of the cytoplasmic tail of AQP3 with that of AQP4 (AQP434) induced inclusion of AQP434 into AQP3 containing vesicular carriers.

Although mechanisms involved in sorting some classes of basolateral membrane proteins are known, others are not. There are a variety of basolateral sorting signals, and the majority of which contain either a tyrosine or a dileucine motif [19]. The medium subunit of the clathrin adaptor protein μ1B interacts with tyrosine motifs [20], [21]. However, sorting of some basolateral proteins containing tyrosine motifs is not μ1B-dependant (e.g., transferrin receptor) [22]. Recently, it was shown that not only AP-1B, but also AP-1 are involved in sorting and AP-1B was able to compensate for the loss of AP-1A [23]. Some basolateral proteins, such as Na/K-ATPase, do not have a *bona fide* basolateral sorting motif [24], [25]. Ankyrin seems to play a role in trafficking of E-cadherin to the plasma membrane, although ankyrin does not bind to the region of E-cadherin containing the basolateral sorting motif [26]. The complexity of sorting signals and different adaptor protein complexes may provide a molecular basis for sorting of some basolateral proteins into common or different carriers based on their sorting signal.

Our results also indicate that differences in the protein content of TGN carriers are maintained during their transport and delivery to the plasma membrane. Spinning disk confocal live-cell microscopy showed that vesicular carriers with the same content of plasma membrane proteins that exit the TGN together are also delivered to the plasma membrane together. Whether this reflects direct delivery from the TGN to the plasma membrane, or an intermediate step through the recycling endosome remains unclear. While we detected some overlap between AQP3-GFP and GFP-AQP4 and transferrin-loaded recycling endosomes, this may reflect the passage of proteins cycling from the pool at the plasma membrane, or trafficking of newly-synthesized AQP3-GFP and GFP-AQP4 from the TGN. Regardless, the fact that AQP3 and AQP4 leave the TGN in separate vesicles and arrive separately at the plasma membrane indicates that if they transit the recycling endosome they must be resorted into separate vesicles for delivery to the plasma membrane. Such a resorting mechanism in the recycling endosome likely involves the same mechanisms that operate in the TGN [27], and hence the difference in sorting information between AQP3 and AQP4 may function in either the TGN or recycling endosome.

Sorting of basolateral proteins into different vesicle carriers in the TGN may be important for cellular regulation of homeostasis in response to physiological signals. The levels of plasma membrane proteins can be regulated by a variety of mechanisms. These include changes in transcription, mRNA stability, protein degradation rates and internalization from the plasma membrane. Our results indicate that the TGN may function as an additional checkpoint by regulating the protein content of vesicle carriers exiting the TGN. For example, the amounts of AQP3 and AQP4 in the basolateral plasma membrane of kidney collecting duct principal cells [8], [9] are regulated independently. Upon long term stimulation with the antidiuretic hormone vasopressin, the plasma membrane level of AQP3 is up-regulated [8], [13] whereas the level of AQP4 is not [13]. While there is increased transcription of both AQP3 and AQP4 that results in more newly synthesized AQP3 and AQP4 entering the TGN, only plasma membrane levels of AQP3 increase [28]. The independent sorting of AQP3 into TGN carriers not containing AQP4 could be essential for maintaining differences in plasma membrane content of these proteins. These studies were performed in cultured kidney epithelial cells, and ultimately it will be important to show how these proteins are separated in the exocytic pathway in kidney epithelia. However, at this time this analysis is beyond the scope of the present studies.

## Materials and Methods

### Constructs and stable cell lines

MDCK IIG cells [29], [30] were transfected with either AQP3-EGFP, EGFP-AQP4 (gifts from Dr. Anita Aperia, Karolinska Institutet, Sweden) or a combination of AQP3-EGFP and Orange-AQP4 using Effectene (Qiagen) and selected with G418 (InVitrogen). Single colonies of cells stably expressing AQP3-EGFP, EGFP-AQP4 or a combination of AQP3-EGFP and Orange-AQP4 were selected. All constructs were stably expressed in MDCK IIG cells without any apparent change in phenotype. Proper expression of constructs was confirmed by immunofluorescence microscopy and Western blotting. For transient transfections, MDCK GII cells or MDCK cells stably expressing VSV-G-YFP (a gift from Dr. Kai Simons) were transfected using Effectene (Qiagen) with combinations AQP3-CFP, CFP-AQP4, or LDL-R-CFP (a gift from Dr. Enrique Rodriguez-Boulan), and Orange-AQP4 and GFP-AQP434 (see below). AQP3-CFP, Orange-AQP4, and CFP-AQP4 was generated by subcloning of AQP3 and AQP4 into Clontech vectors containing CFP or a pEGFP-C1 Clontech vector constructed to contain Orange (a gift from Dr. Roger Tsien) instead of EGFP. Tagging AQP4 with Orange did not change the cellular localization of the protein compared to EGFP-tagged AQP4. To generate a chimeric construct consisting of the cytoplasmic domains of AQP4 fused to the plasma membrane spanning domain of AQP3, a 2-step PCR protocol was used (Figure S7A). First, the three domains (the two cytoplasmic domains of AQP4 and the plasma membrane spanning region of AQP3) were amplified using the following primers and templates:

AQP4–5′-BglII: ATCAGATCTGGGGAAGGCATGAGTGACAGAC

AQP43-3′: CCCCAGGCACTCGGCGACTGCTTTCCAGAAAG

Template EGFP-AQP4

AQP43-5′: CTTTCTGGAAAGCAGTCGCC GAG TGC CTGGGG

AQP34-3′: ACAGAAGACATACTCGCCGATCATCAGCTG

Template AQP3-EGFP

AQP34-5′: CAGCTGATGATCGGCGAGTATGTCTTCTGT

AQP4-3′-KpnI: ACCGGTACCTCATACTGAAGACAATACCTCTCCAGATTGGT

Template EGFP-AQP4

The primers contained overhangs so that the three fragments would adhere in the following PCR. The three products were then pooled and used as template for the 2nd round of PCR where AQP4–5′-BglII and AQP4-3′-KpnI primers were used to amplify the full AQP434 fragment. The product was then cloned into TOPO-pcDNA3.1-GFP-CT (InVitrogen).

### TGN accumulation of proteins, labeling and microscopy

For localization of basolateral proteins in the TGN and in post-Golgi carriers using deconvolution microscopy, cells were seeded on collagen-coated coverslips and allowed to attach and spread for 1 hour. The media was changed to imaging media [phenol-red-free DMEM media (Sigma-Aldrich) with 10% fetal bovine serum (Atlas Biologicals)], 25 mM HEPES (Invitrogen) and cycloheximide (0.02 μg/ml)) and the temperature was switched to 19°C for 2 hours, fixed and stained with Golgi58 K antibody (Sigma) or followed by a 10 min incubation at 37°C. To test if AQP3-EGFP and EGFP-AQP4 co-localized with recycling endosomes, cells were seeded on collagen-coated coverslips and allowed to attach and spread for 1 hour, then the media was changed to serum-free media. After 30 min incubation in serum-free media at 37°C, 0.1 mg/ml Texas-Red transferrin was added and cells were incubated an additional 30 min at 37°C to allow transferrin uptake. The media was then changed to imaging media and the temperature was switched to 19°C for 2 hours followed by a 10 min incubation at 37°C.

For deconvolution microscopy, cells were fixed with 2% paraformaldehyde and cover-slips were mounted on glass slides using Vectashield (vectorlabs). Cells were imaged using a DeltaVision deconvolution microscope system (Applied Precision Inc.) equipped with a Princeton HQ CoolSNAP interline cooled CCD camera. Images were captured using a 60x (NA 1.40) oil objective and a 1.5 optovar and processed using DeltaVision deconvolution software (Applied Precision) on a workstation (Silicon Graphics, Inc.) at the Stanford Medical Center Cell Sciences Imaging Facility. The deconvolution software is automatic without manual manipulation and was performed on both channels; the only manual changes were to make the brightness and contrast the same in both channels. The threshold fluorescence intensity for data acquisition and processing was kept low in order not to exclude weak fluorescence signals. Nevertheless, we cannot exclude the possibility that some weak signals were excluded, but it is unclear whether this would affect the conclusions as those signals may or may not have overlapped.

Images were analyzed using Slidebook (Intelligent Imaging Innovations) or ImageJ [31]. Pearson's coefficient for the Golgi region was calculated after background subtraction using the “Intensity Correlation Analysis” plugin for ImageJ (created by Tony Collins, Wright Cell Imaging Facility, Toronto, Canada and Elise Stanley, C&MB, TWRI, Toronto, Canada). Available: http://www.uhnresearch.ca/facilities/wcif/imagej/colour_analysis.htm#coloc_ica. Accessed 2013 August 8^th^. Pearson's values are indicated as average ± standard error of mean.

To study the localization of AQP3-EGFP and Orange-AQP4 in polarized cells, the cell line stably expressing both proteins was seeded in low calcium (5 µM) media at high density on filters. One hour after seeding, the media was changed to regular media containing 1.8 mM calcium. To study GFP-AQP434 localization in polarized cells, cells transiently expressing GFP-AQP434, cells were seeded on filters. Cells were maintained for 3 days on Transwell filters (Costar), blocked for 2 hours at 19°C, fixed and imaged using an upright LSM510 confocal laser scanning microscope (CLSM; Carl Zeiss, Jena, Germany) and a HCX PL APO 63x (NA 1.20) W Corr CS objective or a Zeiss Axiovert 200 M microscope equipped with a Stanford Photonics, Inc. Mega-10 S30 ICCD camera and Metamorph software.

To study localization of untagged AQP3 and AQP4, MDCK IIG cells [29], [30] were transfected with a combination of AQP3-IRES-EGFP and AQP4-IRES-EGFP (gifts from Dr. Anita Aperia, Karolinska Institutet, Sweden) using Lipofectamine 2000. 48 hrs following transfection, 25 mM HEPES (Invitrogen) and cycloheximide (0.02 μg/ml)) was added and the temperature was switched to 19°C for 2 hours followed by a 10 min incubation at 37°C. Cells were subsequently fixed and stained with anti-AQP3 and anti-AQP4 antibodies as follows: Cells were fixed for 10 minutes in 4% paraformaldehyde, washed in PBS and then permeabilized in 0.1% Triton X-100 and 3% BSA in PBS for 10 minutes. Cells were labeled with primary anti-AQP3 antibody (a gift from the Water and Salt Research Center, AU, Denmark) diluted 1∶100 in 3% BSA in PBS for 2 hours at room temperature. Then coverslips were washed in PBS three times and labeled with secondary Dylight 549 anti-rabbit antibody raised in donkey diluted 1∶500 for 45 min at room temperature in dark. Before labeling with AQP4 antibody, the slides were washed three times in PBS and then blocked in 10% rabbit serum in PBS (Dako). The stain for AQP4 was done similarly, anti-AQP4 primary antibody (Alomone labs, a gift from the Water and Salt Research Center, AU, Denmark) was diluted 1∶3000 and the secondary antibody used was Dylight 649 donkey anti-rabbit. Finally the slides were washed in PBS and mounted on glass slides using mounting medium (Dako). To test whether the rabbit serum block between the two primary antibodies worked, the second primary antibody was omitted before adding the second secondary antibody. This showed that no labeling occurred, and hence the block was sufficient to avoid binding of the second secondary antibody to the first primary antibody. Antibody labeled cells were imaged by laser scanning confocal microscopy with a Zeiss 510 LSM using a 63 x oil Plan Apo objective and a 543 HeNe laser/633 HeNe laser for excitation of Dylight 549 and 649 antibodies. Detection was done by using a longpass 560 emission filter for Dylight 549. For detection of Dylight 649 the Zeiss LSM meta detection system was used (675–700 nm).

For live cell spinning disk microscopy, MDCK IIG cells were seeded in 3 cm MatTek glass bottom dishes, and after 1 hour the cells were subjected to temperature block and release as described above before imaging. Spinning disk microscopy was performed using a Nikon Eclipse Ti microscope equipped with a Yokogawa CSU-X1 spinning disk unit, Andor Laser launcher and an Andor iXon + EMCCD for detection. Imaging was performed using the 491 and 561 laser lines for excitation. Through a Cairn Research Optosplit II color splitter the emission was split with a 550 dichroic mirror and 535 and 605 emission filters (Chroma). The integration time was 40 ms and a 60x Plan Apo oil objective (NA 1.40) was used. Imaging was performed in TIRF like mode with focus set close to the plasma membrane. Image sequences were analyzed manually for vesicle structures.

Spinning disk microscopy was also used to image fixed slides of MDCK cells stably expressing AQP3-EGFP and AQP4-Orange. The same lasers as above were used and emission filters 535/22 and 593/40 were used for detection of EGFP and orange respectively.

## Supporting Information

Figure S1 **Representative example of a single plane of an image of a single cell stably expressing AQP3-EGFP and Orange-AQP4 following a 2** **hour temperature block at 19°C without release at 37°C.** The cells were stained with the Golgi marker G58 K. Overlay shows AQP3-EGFP in green, Orange-AQP4 in red and G58 K staining in blue. Scale bar is 10 μm.
(TIF)

Figure S2 **An example of the effects of deconvolution processing on images.** Images from Figure 5 showing a single plane of non-deconvolved (raw image) and the corresponding deconvolved images of single cells loaded with Texas-Red transferrin and stably expressing AQP3-EGFP 10 minutes following release from a 19°C temperature block. Inserts 1 and 2 show magnified regions from the TGN. Scale bars are 5 μm; 0.5 μm (inserts).
(TIF)

Figure S3 **Representative example of a single plane of a spinning disk confocal microscopy image of a single cell stably expressing AQP3-EGFP and Orange-AQP4 10** **inutes following release from a 19°C temperature block and inserts of the marked areas: arrows point to AQP3-EGFP containing TGN and post-Golgi carriers, arrowheads point to Orange-AQP4 containing TGN and post-Golgi carriers.** Scale bars are: 5 μm (overlay and single channel); inserts: 5 μm.
(TIF)

Figure S4 **Representative example of a single plane of a deconvolved image of a single cell stably expressing AQP3-EGFP and Orange-AQP4 0** **minutes following release from a 19°C temperature block.** Top panel shows the xy plane, bottom panel the z plane through the stacks. Scale bar is 5 μm.
(TIF)

Figure S5 **Representative example of a single plane of a deconvolved image of a single cell stably expressing AQP3-EGFP and Orange-AQP4 0** **minutes following release from a 19°C temperature block and inserts of the marked areas: green arrows point to AQP3-EGFP containing post-Golgi carriers, red arrows point to Orange-AQP4 containing post-Golgi carriers.** Scale bars are: 5 μm (overlay and single channel); inserts: 0.5 μm.
(TIF)

Figure S6 **Representative examples of an image captured by confocal microscopy of polarized cells grown on filters for 3 days stably expressing AQP3-EGFP (green) and Orange-AQP4 (red) immediately after a 2** **hour 19°C temperature block.** A is the xy plane. B is the Z plane at the white line in A. Arrows point to intracellular structures. Scale bars are 10 μm.
(TIF)

Figure S7 (A) Scheme of the PCR procedure used to generate the chimeric construct AQP434 and of the structure of the generated chimera AQP434. The cytoplasmic domains of AQP4 are fused to the 1^st^ and 6^th^ transmambrane domains of AQP3. (B) Confocal images of MDCK cells expressing GFP-AQP434 in cells allowed to polarize 3 days on filters.
(TIF)
