# Supplementary figures and images for: Aquaporin-3 and Aquaporin-4 Are Sorted Differently and Separately in the Trans-Golgi Network
Source: PLoS One. 2013 Sep 18;8(9):e73977. doi: 10.1371/journal.pone.0073977 (PMC3776795; doi:10.1371/journal.pone.0073977)

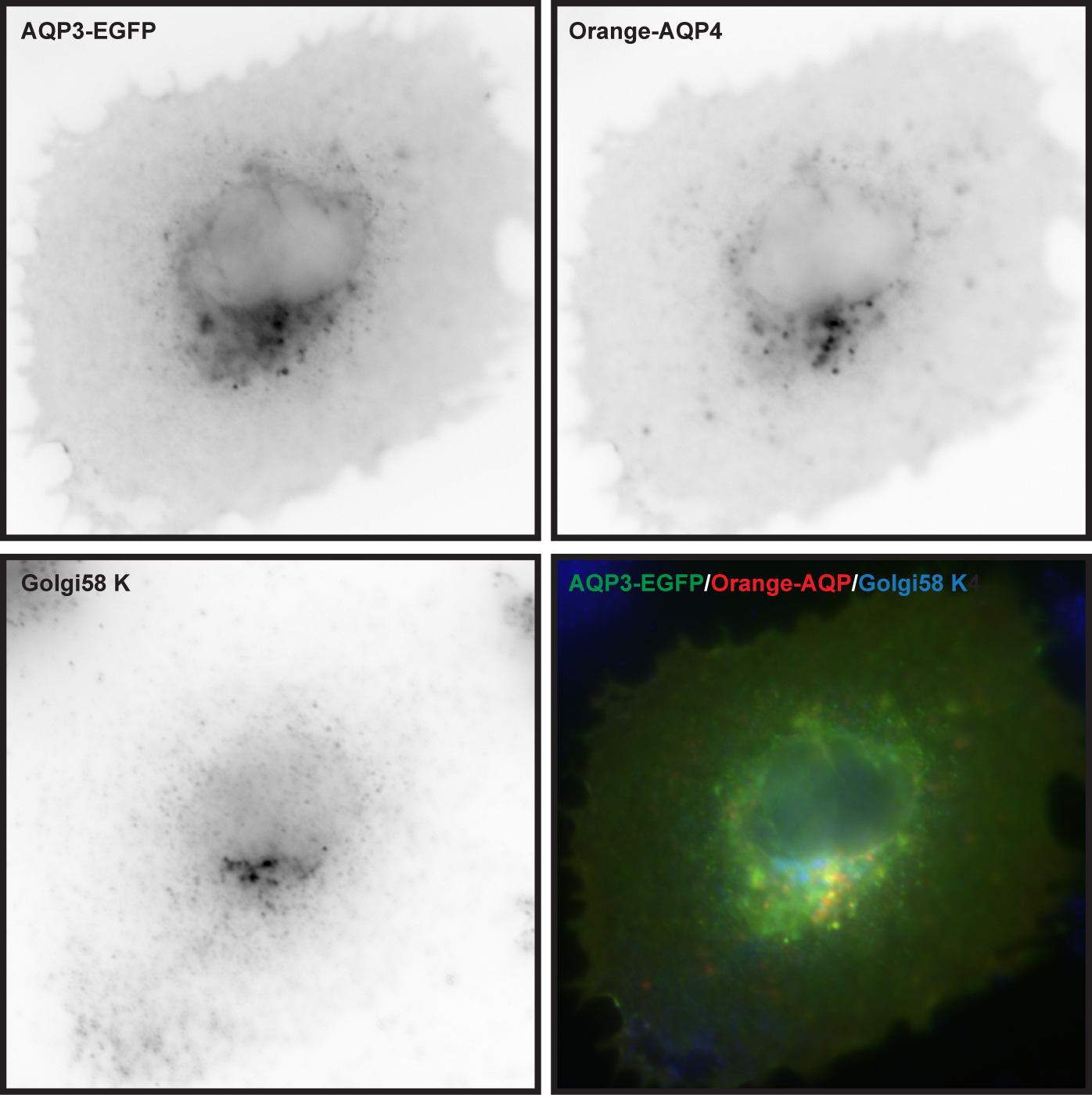

Supplement: Figure S1 — Representative example of a single plane of an image of a single cell stably expressing AQP3-EGFP and Orange-AQP4 following a 2 hour temperature block at 19°C without release at 37°C. The cells were stained with the Golgi marker G58 K. Overlay shows AQP3-EGFP in green, Orange-AQP4 in red and G58 K staining in blue. Scale bar is 10 μm. (TIF) [file pone.0073977.s001.tif]

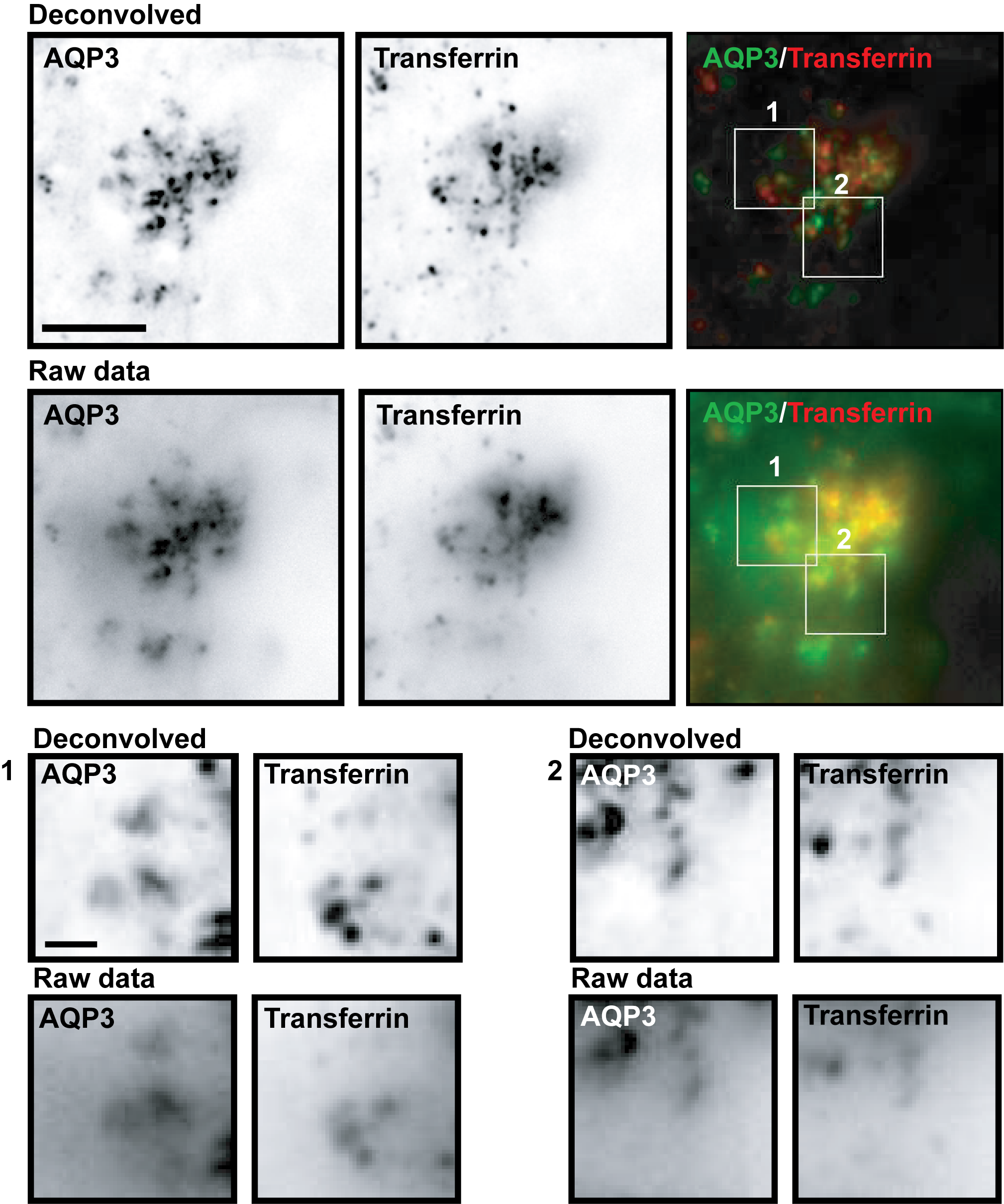

Supplement: Figure S2 — An example of the effects of deconvolution processing on images. Images from Figure 5 showing a single plane of non-deconvolved (raw image) and the corresponding deconvolved images of single cells loaded with Texas-Red transferrin and stably expressing AQP3-EGFP 10 minutes following release from a 19°C temperature block. Inserts 1 and 2 show magnified regions from the TGN. Scale bars are 5 μm; 0.5 μm (inserts). (TIF) [file pone.0073977.s002.tif]

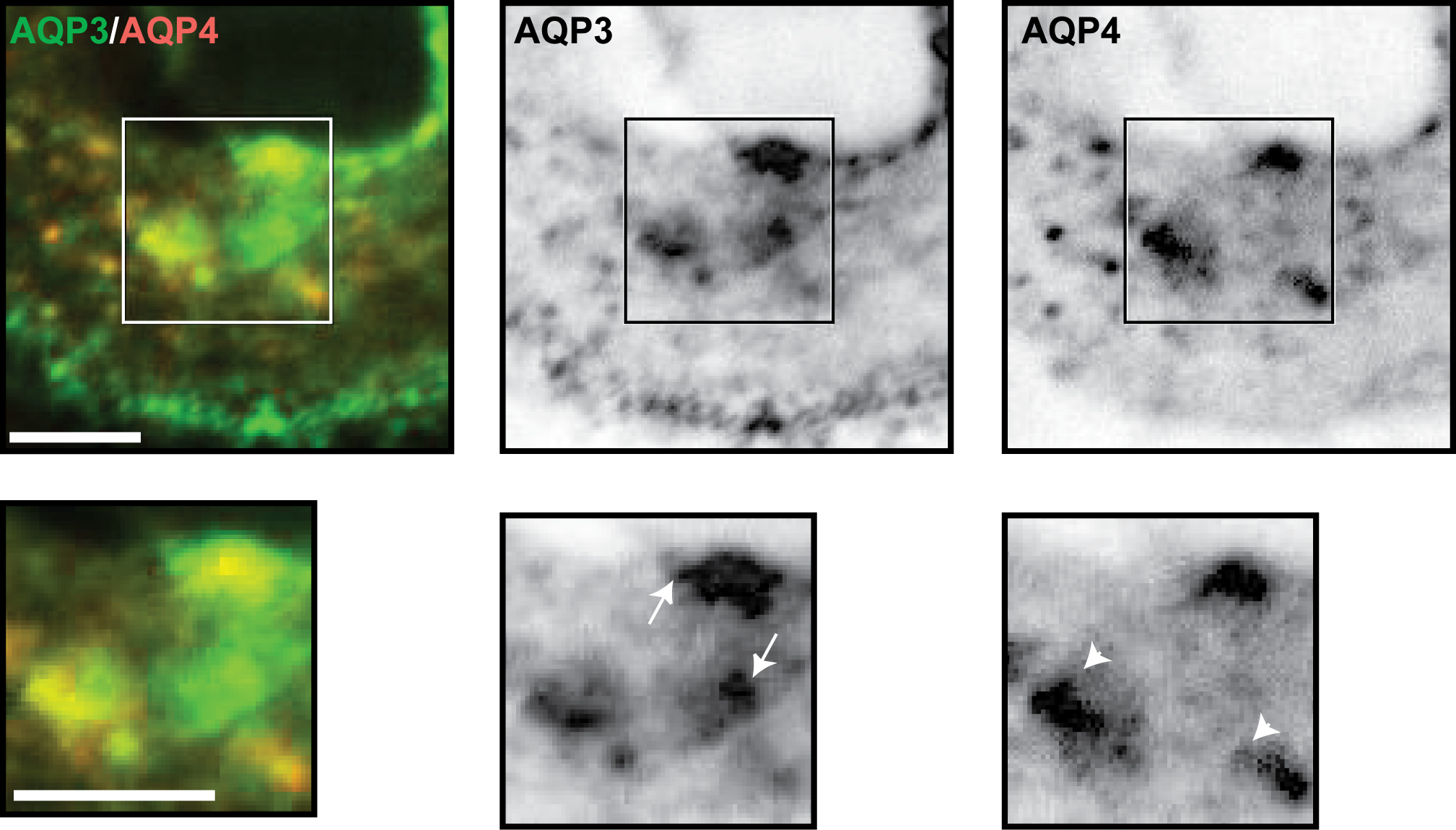

Supplement: Figure S3 — Representative example of a single plane of a spinning disk confocal microscopy image of a single cell stably expressing AQP3-EGFP and Orange-AQP4 10 inutes following release from a 19°C temperature block and inserts of the marked areas: arrows point to AQP3-EGFP containing TGN and post-Golgi carriers, arrowheads point to Orange-AQP4 containing TGN and post-Golgi carriers. Scale bars are: 5 μm (overlay and single channel); inserts: 5 μm. (TIF) [file pone.0073977.s003.tif]

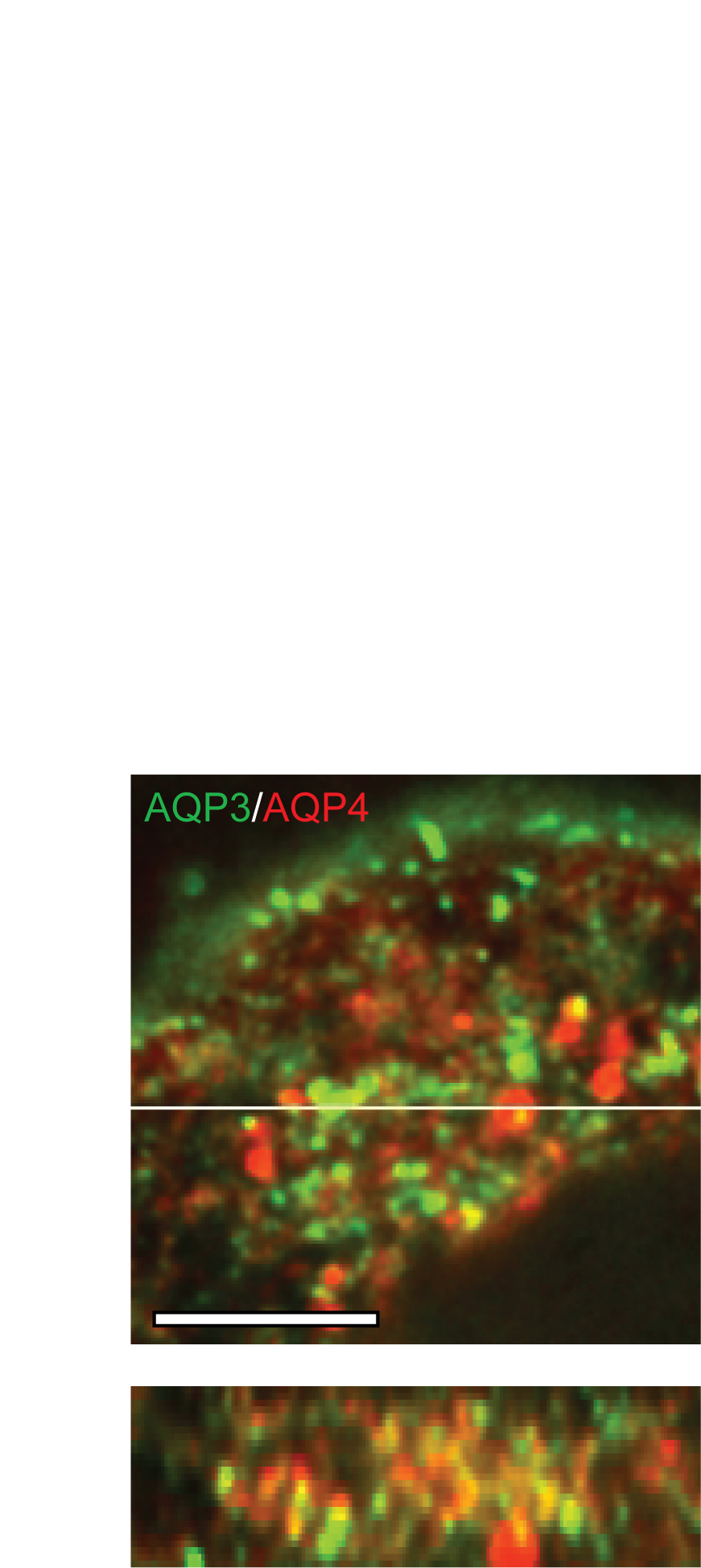

Supplement: Figure S4 — Representative example of a single plane of a deconvolved image of a single cell stably expressing AQP3-EGFP and Orange-AQP4 0 minutes following release from a 19°C temperature block. Top panel shows the xy plane, bottom panel the z plane through the stacks. Scale bar is 5 μm. (TIF) [file pone.0073977.s004.tif]

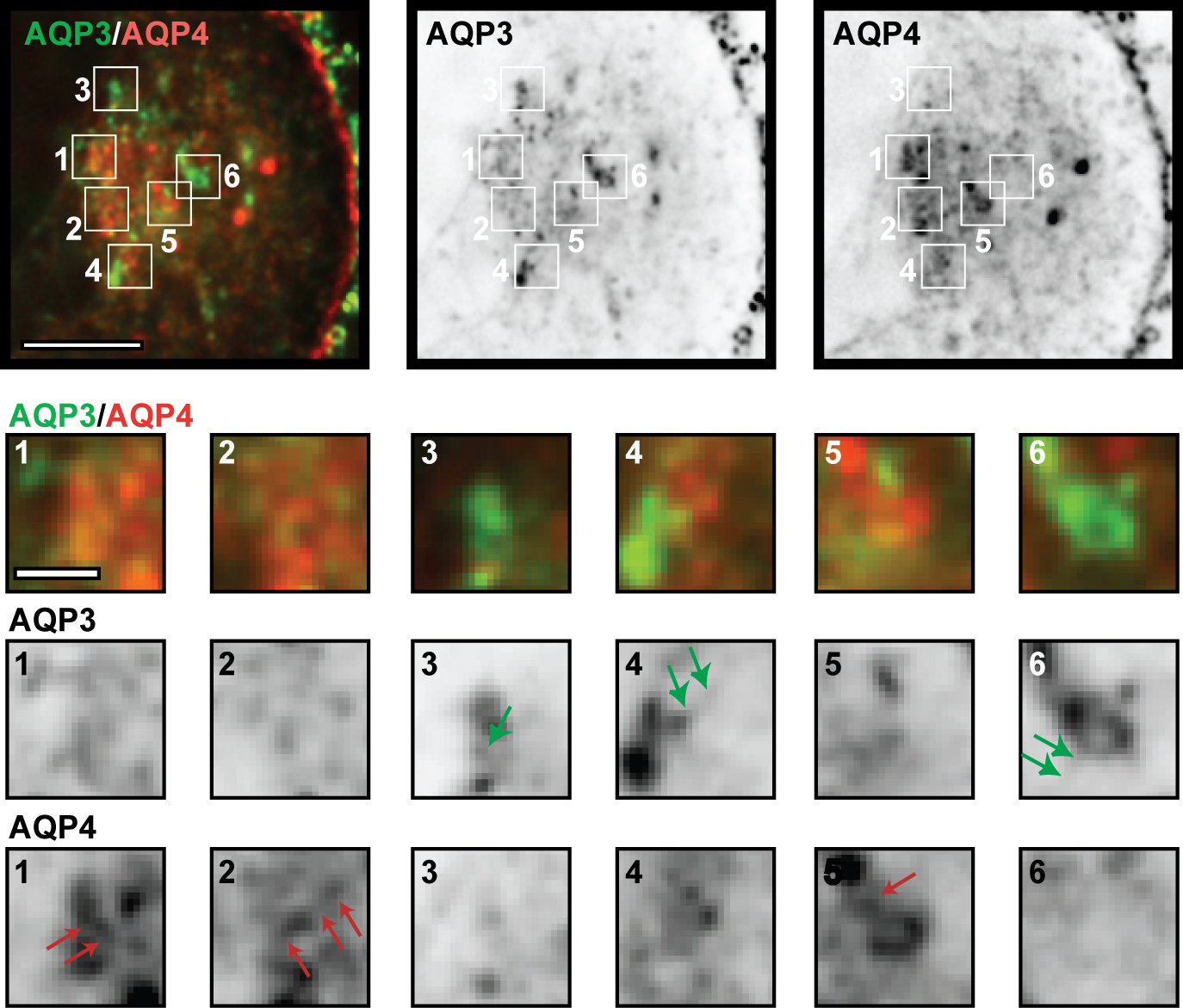

Supplement: Figure S5 — Representative example of a single plane of a deconvolved image of a single cell stably expressing AQP3-EGFP and Orange-AQP4 0 minutes following release from a 19°C temperature block and inserts of the marked areas: green arrows point to AQP3-EGFP containing post-Golgi carriers, red arrows point to Orange-AQP4 containing post-Golgi carriers. Scale bars are: 5 μm (overlay and single channel); inserts: 0.5 μm. (TIF) [file pone.0073977.s005.tif]

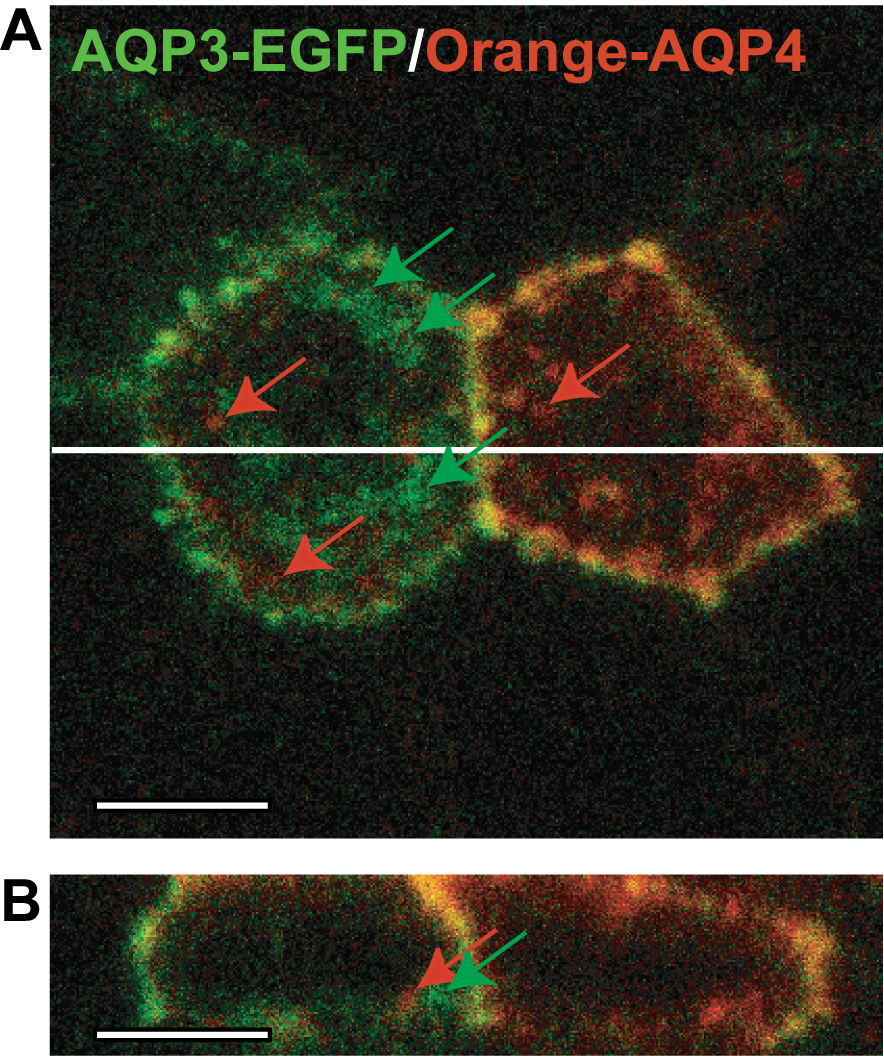

Supplement: Figure S6 — Representative examples of an image captured by confocal microscopy of polarized cells grown on filters for 3 days stably expressing AQP3-EGFP (green) and Orange-AQP4 (red) immediately after a 2 hour 19°C temperature block. A is the xy plane. B is the Z plane at the white line in A. Arrows point to intracellular structures. Scale bars are 10 μm. (TIF) [file pone.0073977.s006.tif]

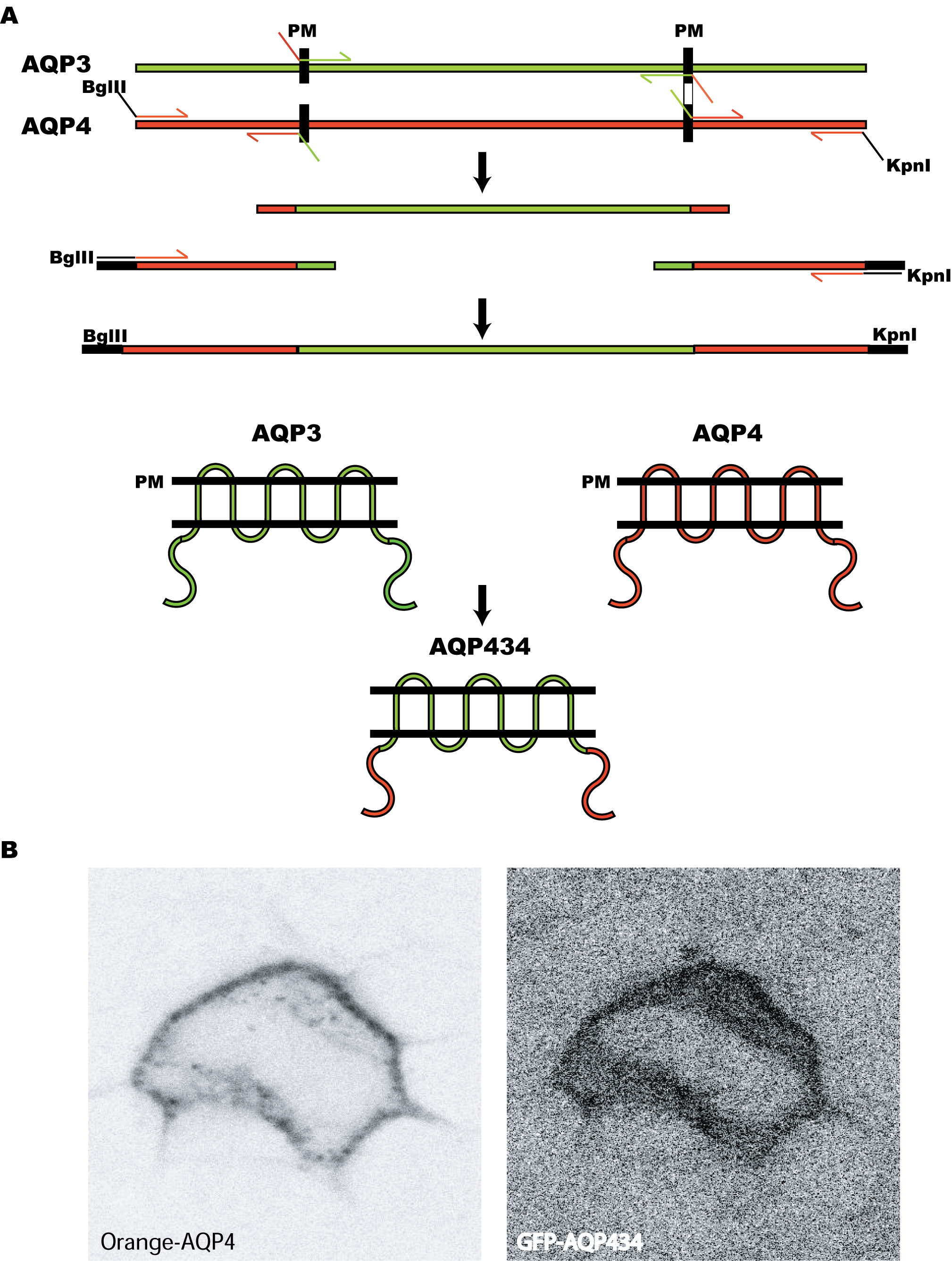

Supplement: Figure S7 — (A) Scheme of the PCR procedure used to generate the chimeric construct AQP434 and of the structure of the generated chimera AQP434. The cytoplasmic domains of AQP4 are fused to the 1st and 6th transmambrane domains of AQP3. (B) Confocal images of MDCK cells expressing GFP-AQP434 in cells allowed to polarize 3 days on filters. (TIF) [file pone.0073977.s007.tif]
